# Holiday Weight Change in a US College Student Sample: A Prospective Observational Cohort Study

**DOI:** 10.1002/osp4.70035

**Published:** 2025-01-08

**Authors:** Hannah B. Yoo, Casen Bigham, Tanisha Basu, Sharmin Akter, Tiffany Tsai, Alexis Brown, Sara Kiros, Shruthi Durai, Claire Brown, Martin Binks

**Affiliations:** ^1^ Department of Psychological Sciences Texas Tech University Lubbock Texas USA; ^2^ Department of Nutritional Sciences Texas Tech University Lubbock Texas USA; ^3^ Department of Radiology Research College of Medicine University of Cincinnati Cincinnati Ohio USA; ^4^ Department of Psychology Princeton University Princeton New Jersey USA; ^5^ College of Education University of Oregon Eugene Oregon USA; ^6^ Department of Biological Sciences Texas Tech University Lubbock Texas USA; ^7^ College of Visual and Performing Arts Texas Tech University Lubbock Texas USA; ^8^ Department of Nutrition and Food Studies George Mason University Fairfax Virginia USA

**Keywords:** college students, holiday weight gain, obesity, overweight

## Abstract

**Objective:**

The November through January holiday period is often thought to produce weight gain, coined “holiday weight gain.” While this trend has been documented among early to midlife adults, it is less certain whether college students experience similar levels of weight gain during this period, as they undergo lifestyle changes (e.g., returning to their families of origin) that may differ from adults at later stages of development. Thus, the primary aim of the current study was to determine body weight and body composition changes during the holiday season in college students. The secondary aim was to examine the association of psychosocial variables with primary outcomes.

**Methods:**

Participants included 47 undergraduate students. Body weight, body composition, and psychosocial variables were measured prior to Thanksgiving break and after returning from December to January break. Differences in body weight and body composition variables were determined using paired‐sample *t*‐tests. Associations of psychosocial variables with changes in primary outcomes were determined by Spearman's correlation coefficients and linear regressions.

**Results:**

On average, college students experienced a 1.08% increase in body weight between study visit 1 in November and study visit 2 in January (0.74 ± 1.81 kg; *p* = 0.02). Lean mass increased (1.49 ± 3.21 kg; *p* = 0.01), while body fat % did not change (*p* = 0.12). Psychosocial variables were not significantly associated with or predictive of changes in body weight or body fat % (*p*'s > 0.05).

**Conclusions:**

College students gained a small, yet significant, amount of weight over the holiday period. Weight gain occurred alongside increases in lean mass but not body fat. Results provide important context to previous findings observing weight gain in college students and adults over the holiday season. Specifically, findings indicate a healthy increase in lean mass—a potential promotive factor in optimal health and wellbeing.

## 1 Introduction

College years may be a critical period for overweight and obesity development, as students face novel lifestyle challenges and frequently report lack of exercise and dietary behaviors inconsistent with recommended nutrition guidelines [1]. Furthermore, weight gained in early adulthood is often not lost and contributes incrementally and substantially over time to weight status and health outcomes in later adulthood [2]. The November through January holiday period is often thought to produce significant weight gain, coined “holiday weight gain” [3]. This is attributable to the fact that regardless of one's particular customs, the season typically brings disruptions in normal routines and introduces other potential contributors to changes in health behavior (e.g., time constraints, stress).

Previous research in adults has shown that while perhaps not as large as the public narrative might imply, adults on average do gain meaningful amounts of weight during the November through January holiday period in the United States. A comprehensive and well‐controlled (single blind) prospective study in adults (*M* age = 34 years old) reported that on average participants had a small, statistically significant increase in body weight (0.37 kg) in the period from November through January [3]. Increases in self‐reported hunger and reduced physical activity appeared to contribute to weight gain. Those who experienced major weight changes over the holiday period (defined as ≥ 2.27 kg) were more likely to be in overweight or obese BMI categories. Notably, participants did not lose the gained weight at 1‐year follow up, suggesting that the additive impacts over a period of decades could account for a significant portion of weight gained during the adult lifespan.

A recent longitudinal study found that adults with obesity (*M* age = 37 years old) experienced an increase of 0.41 kg in body weight during the holiday period (i.e., mid‐November to early January; [4]). Changes in eating habits (i.e., greater instances of restaurant eating) and lowered post‐meal satisfaction were significant contributors to weight gain during the holiday period, whereas total energy expenditure (TEE) was not. Measures of seasonal affective disorder and salivary cortisol (i.e., biomarker for stress) did not significantly change during the holiday period, suggesting that they did not influence weight gain during this period. Although this study may provide further insights into holiday weight changes among adults using objective measurements, unlike the Yanovski et al. study [3], participants in this study were not blinded to the purpose of the study, which may bias the outcomes toward less weight gain. Furthermore, the results of both studies are not generalizable to a younger college student sample.

Studies examining weight changes over the holiday period in college student samples have been mixed. For example, Wagner et al. found no significant change in body weight or body fat %, as measured by the BodPod, between the period immediately before Thanksgiving Day and a few days following New Year's Day [5]. Similarly, Hull et al. found no significant change in weight among college‐aged students from the 2 week prior to Thanksgiving to the post‐New Year's period [6]. Researchers did note a significant increase in body fat mass as measured by dual energy X‐ray absorptiometry (DXA). Notably, neither study specified that participants were blinded to the study's purpose, which could potentially influence college students' behaviors during the holiday period. A subsequent investigation in which participants were blinded to the purpose of the study found that college students experienced significant increases in body weight (0.78 kg), body fat %, and blood pressure between mid‐ November and early January [7]. Increases in body fat %, were greater among those with obesity, and for all participants, exercise did not appear to contribute to weight changes. However, researchers have used bioelectrical impedance analysis to measure body fat %, which has documented limitations in its reliability and precision, especially compared to gold‐standard measurement tools (e.g., DXA, BodPod; [8]).

The holiday season and resulting “holiday weight gain” may not be comparable between college students and adults for several reasons. For example, college students are adapting to independent lifestyles during the school semester (e.g., navigating academic pressures, increased time demands, challenges of independent living and or cohabitating with roommates, and challenges of early financial responsibility). During the holiday period, many return to circumstances more akin to their “pre‐college lifestyle” as they return home and stay with their families. Thus, the psychosocial precursors (e.g., final exams) to college students' holiday periods, as well as the experience of returning to and being at home, may look markedly different than for working, midlife, or older adults. Furthermore, the COVID‐19 pandemic may have exacerbated these stressors, as many students reported difficulties in relocation (i.e., returning back to their family home) characterized by disruptions in their adult identity and sense of autonomy [9]. Taken together, it is important to consider weight and body composition changes in the context of specific life course events and relevant psychosocial factors.

Although a few well‐controlled studies suggest that adults gain weight over the holiday period, similar studies in college students have *not* consistently replicated this finding. To the authors' knowledge, no studies have been conducted in which college students were explicitly blinded to the primary purpose of the study *and* assessed using gold‐standard tools. Given apparent gaps in the literature, additional well‐controlled studies in college age populations are needed in order to better understand potential changes in weight and body composition, as well as broader psychosocial contributors, during this critical period.

As such, the primary aim of the current study was to determine changes in body weight and body composition during the holiday season in full‐time undergraduate students. The secondary aim was to examine the potential association of psychosocial variables (i.e., perceived stress, depression, anxiety, and social support) with primary outcomes. We hypothesized that there would be a small but significant increase in body weight and body fat %. Additionally, we hypothesized that stress (PSS) and anxiety and depression scores (HADS) would have a positive relationship with weight change, and perceived social support (AESSI) would have a negative relationship with weight change.

## 2 Methods

### 2.1 Subjects

Forty‐seven undergraduate students were recruited from colleges in the Lubbock Texas community. Subjects were recruited via e‐mail and list‐servs, social media, electronic and traditional notice boards, radio and television, word of mouth, and the laboratory's existing recruitment database. Eligibility criteria included being 18–25 years of age, a full‐time student, and currently enrolled in a bachelor's degree program or equivalent. There were no other exclusionary criteria other than not meeting the inclusion criteria. Initial eligibility was assessed via telephone screening and verified at study visit 1 (Baseline Assessment).

### 2.2 Study Timeline

In order to create a more generalizable observation, this study sought to limit the measurement points to just three break periods: (1) immediately prior to the Thanksgiving break (1–2 weeks prior), (2) immediately prior to the December–January break (1–2 weeks prior), and (3) immediately after return from the December–January break (1–2 weeks after) break. Ultimately, due to an unanticipated non‐return of students from Thanksgiving break due to the COVID‐19 pandemic—only the first and last timepoints were observed.

### 2.3 Pre‐ Post‐Assessment Visits

Upon arrival for the pre‐assessment visit, subjects were provided with informed consent, given the opportunity to ask questions, and requested to sign prior to data collection. Subjects were informed that the study's purpose was to understand how factors, such as stress and social support, influence undergraduate health and lifestyle. As such, they were blinded to the primary objective of weight and body composition change to minimize behavior change associated with expectations around weight loss. Participants were compensated $30 ($15 per visit) in addition to two BodPod body composition assessments, which were provided after the completion of the study.

Anthropometric Measures. Height was measured using a mechanical stadiometer. Blood pressure was assessed using a digital blood pressure monitor (Omron Healthcare Inc., IL, USA Digital BP Monitor HEM‐907XL). Two readings were taken in immediate succession automatically and the average of the two readings was calculated by the device and recorded. Participants' weight and body composition were measured using BodPod (Cosmed USA Inc., Concord, CA), which uses air displacement plethysmography to provide reliable and accurate assessments of fat and lean mass [10]. Prior to study visits 1 and 2, participants were asked to refrain from exercise and to fast within the 2‐h period before the visit. They confirmed adherence to the instructions before measurements.

Anxiety and depression. The Hospital Anxiety and Depression Scale (HADS) is a commonly used 14‐item measure that assesses participants' anxious distress and depressive symptoms over the past week [11]. Seven items assess for depressive symptoms (e.g., *I feel as if I am slowed down*), and seven items assess for anxiety symptoms (e.g., *I feel tense or “wound up”*). Items are rated on a Likert‐type scale and then summed to create a summary score for each subscale, with higher scores indicating higher depressive or anxious distress. HADS has been shown to capture nonclinical levels of anxiety and has demonstrated acceptable‐to‐good internal consistency for both anxiety (Cronbach's alpha = 0.82) and depression subscales (Cronbach's alpha = 0.73) in a college student sample [12].

Perceived stress. Participants' levels of stress were assessed using the 10‐item Perceived Stress Scale (PSS; 14). Items are rated on a Likert‐type scale from 0 (*Never*) to 4 (*Very Often*). Items 4, 5, 7, and 8 are reverse coded and then summed to create a total score. The PSS demonstrated good internal consistency (0.84–0.86) in the original college samples used for scale development [13].

Social support. The Alford Edwards Social Support Inventory (AESSI) is a validated self‐report measure of satisfaction with one's support from various sources including one's spouse/partner, family members, friends, co‐workers/colleagues, social contacts, and healthcare providers [14]. The AESSI assesses four forms of social support: emotional, instrumental, informational, and comparison. Only items assessed for emotional support were summed to create an emotional support index score, reflecting participants' satisfaction with how well others listened to, understood, and cared for them. Prior studies have demonstrated excellent internal consistency for the AESSI (Cronbach's alpha = 0.93; [15]).

### 2.4 Data Analytic Plan

Given recommendations for missing data in small sample sizes, analyses were run only on completed cases [16]. All data analyses were conducted using the Statistical Package for Social Sciences Version 29 (SPSS, Armonk, New York; [17]). Descriptive statistics including means and standard deviations for body weight, body composition (i.e., body fat %, lean mass), BMI, blood pressure (i.e., SBP and DBP), and psychosocial measures (i.e., HADS, AESSI, PSS) at both pre‐holiday (visit 1) and post‐holiday (visit 2) were calculated. Two‐tailed paired *t*‐tests were used to analyze changes in body weight, body composition, and psychosocial variables from visits 1 to 2 (Research Aim 1). A Spearman's rank‐order correlation matrix was created to identify potentially meaningful correlations among psychosocial variables at baseline, as well as between psychosocial variables and anthropometric measures (i.e., body weight and body composition).

Exploratory multiple linear regressions were used to examine selected psychosocial variables at baseline as predictors of change in body weight and body fat % (Research Aim 2). Linear regressions were also used to examine any significant changes in psychosocial variables as predictors of body weight or body fat percentage change. Multicollinearity of psychosocial variables was assessed using variance inflation factors (VIFs), excluding variables with VIFs above 10. Selection of psychosocial measures was based on prior literature implicating stress and social support as potential contributors to weight gain during college years and holiday periods [18].

### 2.5 Ethics

The TTU Human Research Protection Program and Institutional Review Board approved the study (TTU IRB #2020‐828). All procedures were conducted in accordance with the Declaration of Helsinki amended in 2000 [19]. Informed written consent was obtained from all subjects who met the eligibility criteria.

## 3 Results

Thirty‐four participants completed both study visits (*M* age = 20.26, SD = 1.69). Approximately 79% of the current sample identified as female (*n* = 27) and the remaining 21% identified as male (*n* = 7). At study visit 1, the mean body weight (in kg) of the sample was 64.20 (SD = 14.24), and mean body fat % was 22.29 (SD = 9.74). The mean BMI was in the non‐overweight range at 23.09 kg/m^2^ (SD = 3.81). All other variables (e.g., SBP, DBP, lean mass) for study visits 1 and 2 can be found in Table 1. On average, participants gained 0.74 kg (SD = 1.81), representing a weight increase of 1.08% (SD = 2.76%) from pre‐ to post‐holiday period. Results from two‐tailed paired *t*‐tests indicate significant changes in body weight (*t* (33) = −2.38, *p* = 0.023, 95% confidence interval (CI): 0.11–1.37) but no significant changes in body fat % (*t* (33) = 1.59, *p* = 0.12, 95% CI: −3.68 to 0.45) between the two time points. Mean lean mass increased significantly from study visit 1 to study visit 2 (*M* change = 1.49; *t* (33) = −2.69, *p* = 0.011, 95% CI: 0.36 to 2.51).

**TABLE 1:** Demographic, anthropomorphic, and psychosocial data across study visits.

	Study visit 1	Study visit 2
	*M* (SD) or %	*M* (SD) or %
Age (years)	20.26 (1.70)	—
Sex		
Male (%)	20.6	—
Female	76.5	—
Weight (kg)	64.20 (14.24)	64.94 (14.71)
BMI (kg/m^2^)	23.09 (3.81)	23.51 (3.94)
< 18.5	2.9	5.9
18.5–25	67.6	61.8
25 to < 30	26.5	26.5
30 ≤	2.9	5.9
Body fat (%)	22.29 (9.74)	20.67 (9.33)
Fat mass (kg)	14.86 (8.66)	14.09 (8.50)
Lean mass (kg)	49.34 (9.84)	50.82 (9.72)
Blood pressure		
SBP (mmHg)	116.88 (9.34)	114.41 (10.48)
DBP (mmHg)	73.21 (9.23)	73.53 (7.65)
HADS‐anxiety	9.88 (4.28)	9.44 (3.96)
HADS‐depression	4.24 (3.09)	4.24 (2.98)
AESSI	15.43 (5.28)	14.5 (5.07)
PSS	21.91 (6.62)	19.31 (8.69)

Regarding psychosocial variables (see Table 1), anxiety scores were slightly elevated (mild; *M* = 9.89, SD = 4.28), and depressive symptoms were in the healthy range (*M* = 4.24, SD = 3.09) at baseline. No significant change (pre‐ post‐) was noted for anxiety or depression scores (*M* = 9.44, SD = 3.96, *M* = 4.24, SD = 2.98, *p*'s > 0.05), respectively. Mean emotional support score was 15.43 (range = 5–24, SD = 5.28), and mean perceived stress score was 21.91 (range = 9–34, SD = 6.62) at study visit 1. Perceived stress, but not emotional support, was significantly lower at study visit 2 (*M* change = −2.06; *t* (30) = 2.59, *p* = 0.015).

Spearman's rank‐order correlation tests revealed no significant correlations among any psychosocial variables or anthropometric outcomes (i.e., change in body weight, change in body fat %). Changes in body weight and body fat percentage were significantly correlated with one another (*r* = 0.43, *p* = 0.01). Changes in body fat % and lean mass were inversely correlated with one another (*r* = −0.87, *p* < 0.001). Depression and anxiety scores at baseline were significantly correlated with one another (*r* = 0.41, *p* = 0.02). Anxiety and perceived stress (*r* = 0.70, *p* < 0.001) as well as depression and perceived stress (*r* = 0.58, *p* < 0.001) were strongly correlated at baseline. All correlations can be viewed in Table 2.

**TABLE 2:** Spearman's rank‐order correlation matrix of psychosocial variables at baseline and anthropometric changes.

Measured variable	1	2	3	4	5	6
1. HADS‐anxiety	—					
2. HADS‐depression	0.41*	—				
3. AESSI	−0.13	−0.34	—			
4. PSS	0.70**	0.58**	−0.43	—		
5. Weight change	0.05	0.15	−0.07	0.14	—	
6. Body fat % change	−0.14	0.16	0.03	−0.07	0.43*	—
7. Lean mass change	0.24	−0.05	−0.03	0.21	−0.05	−0.87**

*p < 0.05; **p < 0.01 (two‐tailed).

VIFs for all psychosocial variables were all below the cutoff value of 10, and thus, all selected variables were included in the regression models. Results from exploratory multiple linear regression analyses indicated that none of the psychosocial variables at baseline were significant predictors of change in body weight before (*F* (4) = 1.27, *p* = 0.33) or after (*F* (5) = 1.62, *p* = 0.22) adjusting for baseline body weight. Regression analyses including *only* emotional support and perceived stress as predictors of change in body weight were not significant. Similarly, psychosocial variables did not significantly predict change in body fat percentage before (*F* (4) = 0.31, *p* = 0.86) or after (*F* (5) = 0.36, *p* = 0.87) controlling for baseline body fat percentage. Regression analyses including *only* emotional support and perceived stress as predictors of change in body fat percentage were not significant. Results for all regression analyses can be seen in Table 3.

**TABLE 3:** Multiple linear regressions predicting change in weight and body fat %.

Measure variable	*B*	SE	*p*	95% CIs	*R* ^2^ (%)
Criterion: Weight change					
Unadjusted model					25.2
HADS‐anxiety	0.33	0.17	0.08	[−0.04, 0.70]	
HADS‐depression	−0.09	0.15	0.55	[−0.42, 0.23]	
AESSI	−0.02	0.08	0.79	[−0.19, 0.15]	
PSS	−0.05	0.09	0.59	[−0.25, 0.15]	
Adjusted model					36.7
HADS‐anxiety	0.31	0.17	0.08	[−0.05, 0.66]	
HADS‐depression	−0.08	0.15	0.61	[−0.39, 0.24]	
AESSI	−0.05	0.08	0.52	[−0.22, 0.12]	
PSS	−0.07	0.09	0.48	[−0.25, 0.13]	
Baseline weight	0.06	0.04	0.13	[−0.02, 0.13]	
Criterion: Body fat % change					
Unadjusted model					7.7
HADS‐anxiety	0.61	0.73	0.42	[−0.95, 2.17]	
HADS‐depression	−0.01	0.65	0.99	[−1.39, 1.37]	
AESSI	0.15	0.34	0.66	[−0.57, 0.87]	
PSS	−0.28	0.39	0.49	[−1.11, 0.56]	
Adjusted model					11.4
HADS‐anxiety	0.60	0.74	0.44	[−1.0, 2.19]	
HADS‐depression	−0.02	0.65	0.97	[−1.43, 1.38]	
AESSI	0.19	0.35	0.58	[−0.55, 0.94]	
PSS	−0.22	0.40	0.58	[−1.09, 0.64]	
Baseline body fat %	−0.12	0.16	0.46	[−0.45, 0.22]	

## 4 Discussion

On average, college students in our sample gained a small but statistically significant amount of weight (0.74 kg; 1.08% increase) between the pre‐holiday period (prior to Thanksgiving) and post‐holiday period (mid‐January). This finding is consistent with the prior single‐blinded study among college students [7], resembling patterns similar to those of adult populations [3, 4]. While the magnitude of weight gain is small, it is still noteworthy, as other studies suggest that weight gained during the holidays is not completely lost one‐year follow up. In fact, Yanovski et al. found that the weight gained during the holiday period contributed substantially to the total weight gained over the entire year. In the context of a lifespan, incremental weight gain repeated annually and over many years without subsequent weight loss can become clinically meaningful.

Interestingly, a closer look at body composition between the two time points highlights an interesting, and less concerning, potential driver for such weight gain. Participants in the present study gained a significant amount of lean mass over the holiday period (1.49 ± 3.21 kg), despite no significant changes in body fat %. It is possible that increases in lean body mass reflect greater engagement in physical activity over the holiday period or increases in dietary quality, as college students had breaks from their typical course schedule, and many returned to their families of origin. Given the associations between low or loss of lean body mass and chronic diseases [20] and increased mortality [21], the current pattern of findings does not indicate a growing cause of concern—but rather a potential protective factor—among this group. Future studies with larger samples should investigate potential moderating variables to elucidate who may be at greatest risk for incremental holiday weight gain driven by body fat % versus lean body mass.

Increases in body weight and body fat percentage were not significantly related to students' baseline psychosocial stress, social support, or depressive and anxiety symptoms. Interestingly, students reported significantly lower levels of perceived stress at study visit 2 than at study visit 1, with no changes in perceived emotional support from others. Although post hoc exploratory regression analyses did not find the reductions in students' stress (as indicated by the change score) to be predictive of weight or body fat %, this lack of statistical significance may be limited by the small sample size. If this were the case and the noted change scores were in fact accurate, one potential explanation might be that reductions in stress may have coincided with increases in other health behaviors (e.g., exercising, increasing daily activity) that could explain increases in lean body mass and overall weight. Future research with a larger sample may further elucidate these relationships.

There are some limitations. First, while adequately powered for weight, our primary outcome of interest, other variables such as body fat may not have been adequately powered in this sample. Secondly, the short time frame coupled with the small effect size for body fat % and measurement error of the BodPod [22] may have masked changes that could be better detected using a more precise body fat % measurement such as Dual Energy X‐ray Absorptiometry (DXA). These factors will need to be considered in future studies.

Notably, the current study was unable to control for key variables related to college students' weight, psychosocial well‐being, and health behaviors. For example, there are recognized patterns of weight fluctuations that can affect students at different points in their college years (such as the “Freshman 15” phenomenon). These weight fluctuations may have influenced the findings observed in the present study, as these students may have returned to more normal eating and exercise patterns after returning home for the holidays. Additionally, data collection took place immediately before and after the 2020–2021 holiday season. While the primary university was engaged in the normal academic calendar for the semester preceding the holiday break, some extracurricular or social activity may have been restricted on campus, and some students may have experienced greater restrictions returning back to their home states (although TTU has a high percentage of students from Texas where restrictions were less prevalent during this timeframe).

Furthermore, while we intended to measure the second time point immediately after the Thanksgiving holiday (3 timepoints total), a university‐wide decision to circumvent an uptick in campus COVID‐19 cases prevented students from returning to campus after the Thanksgiving holiday. Although it was only a matter of a few weeks before students completed the remainder of the semester from their family homes, the impact of a slightly a‐typical student holiday schedule and the backdrop of a worldwide pandemic is an important contextual element to be considered in interpreting the results. It is also a valuable anomaly that can inform the literature in two ways: (1) should future larger scale nationally representative studies replicate these preliminary findings, they will add support to this unique positive finding in college students, and (2) if such a study shows this to be unique to the context of the pandemic, our study reports one potential positive outcome related to the experience of college students during this difficult time.

The current study has several strengths and contributes to the literature by comprehensively examining holiday seasonal weight and body composition change, as well as relevant psychosocial contributors, in college age students. Additionally, participants were blinded to the study's purpose to minimize the potential impact of primary outcomes (body weight, body composition). Lastly, while unintended, limited assessment timepoints minimized the likelihood of reactive behavior changes across the observation period.

## 5 Conclusions

Taken together, the present investigation sought to clarify inconsistencies in the extant literature demonstrating incremental holiday weight gain among college student populations while improving upon the methodological limitations of prior studies. A novel and important finding was that lean body mass significantly increased, while body fat percentage did not change. These results provide important context to prior studies observing significant weight gain over the holiday period among adults and challenge interpretations of observed weight gain being prescient of later adulthood obesity. Instead, current findings are suggestive of a healthy increase in metabolically active lean mass—a potential promotive factor in optimal health and wellbeing.

## Author Contributions

H.B.Y. contributed to conceptualization of the research design, conducting and interpreting statistical analyses, and manuscript writing. Ca.Bi. and T.B. participated in conceptualization, data collection, data management, and contributed to writing the manuscript. S.A. was responsible for conceptualization, data collection, and contributed to writing the manuscript. T.T. was responsible for data management and contributed to writing the manuscript. A.B. and S.K. contributed to conceptualization, data management, and writing the manuscript. Cl.Br. contributed to conceptualization, primary data collection, data management, and writing of the manuscript. M.B. was responsible for conceptualization and interpreting results, overseeing data collection and management, and writing the manuscript.

## Conflicts of Interest

The authors declare no conflicts of interest.

## Data Availability

The datasets generated and/or analyzed during the current study are available from the corresponding author on reasonable request.
